# A Comprehensive Evaluation of Xpert MTB/RIF Assay With Bronchoalveolar Lavage Fluid as a Single Test or Combined With Conventional Assays for Diagnosis of Pulmonary Tuberculosis in China: A Two-Center Prospective Study

**DOI:** 10.3389/fmicb.2018.00444

**Published:** 2018-03-13

**Authors:** Xiaofu Pan, Shoufeng Yang, Margaret A. Deighton, Yue Qu, Liang Hong, Feifei Su

**Affiliations:** ^1^Department of Laboratory Medicine, The Third Affiliated Hospital of the Wenzhou Medical University, Rui’an, China; ^2^Department of Infectious Diseases, Wenzhou Central Hospital, Zhejiang, China; ^3^School of Applied Sciences, RMIT University, Bundoora, VIC, Australia; ^4^Department of Laboratory Medicine, The Second Affiliated Hospital and Yuying Children’s Hospital of Wenzhou Medical University, Wenzhou, China; ^5^Biomedicine Discovery Institute, Department of Microbiology, School of Medicine, Nursing and Health Sciences, Monash University, Clayton, VIC, Australia; ^6^Department of Infectious Diseases, The Third Affiliated Hospital of Wenzhou Medical University, Rui’an, China

**Keywords:** pulmonary tuberculosis (TB), diagnosis, Xpert MTB/RIF, smear-microscopy, culture, bronchoalveolar lavage fluid (BALF), performance analysis

## Abstract

**Introduction:** The Xpert MTB/RIF is recommended by the World Health Organization as a first line rapid test for the diagnosis of pulmonary tuberculosis (TB); however, China does not routinely use this test, partially due to the lack of a sufficient number of systematic evaluations of this assay in local patients. The aims of this study were to comprehensively assess the diagnostic performance of Xpert MTB/RIF, either alone or in combination with conventional assays for the diagnosis of pulmonary TB in adult Chinese patients.

**Methods:** Xpert MTB/RIF tests were performed in 190 adult patients with suspected pulmonary TB, using bronchoalveolar lavage fluid (BALF) as test specimens. In parallel, conventional tests were carried out using the same BALF samples. Using two different reference standards, the performance of Xpert MTB/RIF, conventional assays and their combinations were evaluated.

**Results:** Using mycobacterial culture as the reference comparator, Xpert MTB/RIF was found to be superior to smear-microscopy in detecting *Mycobacterium tuberculosis*. When final diagnosis, based on clinical criteria, was employed as the reference standard, Xpert MTB/RIF showed an even higher accuracy of 72.1%, supported by a sensitivity of 61.1% and specificity of 96.6%. Xpert MTB/RIF also demonstrated a powerful capability to identify pulmonary TB cases undetected by culture or smear-microscopy. Combining smear-microscopy and Xpert MTB/RIF was found to be the most accurate early predictor for pulmonary TB. Rifampicin resistance reported by Xpert MTB/RIF slightly deviated from that by phenotypic antibiotic susceptibility testing and requires further study with a larger sample size.

**Conclusion:** This two-center prospective study highlights the value of Xpert MTB/RIF with BALF in diagnosing pulmonary TB in adult Chinese patients. These findings might contribute to the optimization of current diagnostic algorithms for pulmonary TB in China.

## Introduction

Tuberculosis (TB) is a leading global public health problem, with an extremely high mortality if left untreated (WHO, 2017). China is among the six countries that account for 60% of all new TB cases worldwide, sharing 918,000 (8.8%) new cases annually (WHO, 2017). The lungs are still the major site of infection for new and relapsed cases, varying from 54 to 97% of all cases reported in the top 30 high-burden countries (WHO, 2017). Despite the introduction of the WHO End-TB Strategy, the rate of decline in TB globally remained at only 1.5% between 2014 and 2015 (WHO, 2017), underlining an urgent need of better and more efficient implementation of anti-TB strategies.

Misdiagnosis and late diagnosis of TB delay access to appropriate antibiotic treatment and promote disease transmission. One of the major anti-TB strategies endorsed by WHO, also the key to reducing the spread of the epidemic, is to introduce rapid and accurate diagnostics for early TB screening and confirmation. Conventional laboratory methods for diagnosis of infection with *Mycobacterium tuberculosis* include smear-microscopy and mycobacterial culture. Although smear-microscopy and culture have been used as the screening and confirmatory “gold standard” tests, respectively, for decades, they have significant limitations of low-sensitivity, being time-consuming and in some countries, difficult to access due to the lack of suitable laboratory facilities (Detjen et al., 2015). Xpert MTB/RIF (Cepheid Inc., Sunnyvale, CA, United States) is a real-time polymerase chain reaction (RT-PCR) based molecular diagnostic assay and is the only rapid diagnostic test endorsed by WHO for detection of *M. tuberculosis* and multidrug resistance (WHO, 2017). This test is simple to perform, rapid, and accurate for early diagnosis of TB in both adults and children (Bates et al., 2013; Friedrich et al., 2013; Lee et al., 2013; Theron et al., 2013; Detjen et al., 2015; Heidebrecht et al., 2016). China has not officially introduced Xpert MTB/RIF into routine TB diagnosis and management, although it is one of the countries with the highest TB burden (WHO, 2017). This was at least partially due to the lack of a sufficient number of clinical studies to endorse the application of this assay for patients with suspected TB in China. Yin et al. (2014) previously examined the performance of Xpert MTB/RIF combined with bronchoalveolar lavage fluid (BALF) in identifying pulmonary TB in patients under 18 years of age. This age group, however, does not represent the age groups that dominate among the TB patients in China. A recent regional tuberculosis prevalence survey estimated the proportion of younger TB patients aged 0–14 years to be only around 1% (Wu et al., 2017). Ou et al. (2015) assessed the performance of Xpert MTB/RIF in Chinese patients using sputum as the test specimen. Sputum is not considered as the most reliable specimen for TB diagnosis in China and using sputum might not reflect the true performance of Xpert MTB/RIF properly (Hill and Whalen, 2015). A prospective study that systematically assesses this assay, using more reliable respiratory specimens and targeting adult age groups that dominate among the TB patients in China is still needed.

The source of test samples has a significant influence on the performance of all diagnostic assays. Sputum, either spontaneously expectorated or induced, has been often linked to a low diagnostic yield for detecting *M. tuberculosis*, due to insufficient sample quantity and/or quality (Sakundarno et al., 2009; Hepple et al., 2012; Meyer et al., 2017). Although bronchoscopy is considered to be an invasive and costly technique that requires specific training of the infectious disease physician, it has been recommended for patients with suspected pulmonary TB in regions with a high frequency of TB (Iyer et al., 2011), or at least for those who failed to spontaneously produce sputum (Lee et al., 2013). Recent studies have found that when combined with Xpert MTB/RIF, BALF could provide accurate results in detecting early-stage pulmonary TB, in particular in smear-negative patients (Theron et al., 2013).

By designing and carrying out a well-controlled prospective study, we sought to systematically assess the utility of the Xpert MTB/RIF assay as a single test or combined with smear microscopy, using BALF as the test specimen, with mycobacterial culture as the reference standard. In a second evaluation, we used the final diagnosis, based on clinical and laboratory data, as the reference to provide a more comprehensive and reliable standard. We anticipate that this work will provide robust data to optimize the current diagnostic algorithms for pulmonary TB in Chinese adult patients.

## Materials and Methods

### The Two Centers and Overall Study Design

**FIGURE 1:**
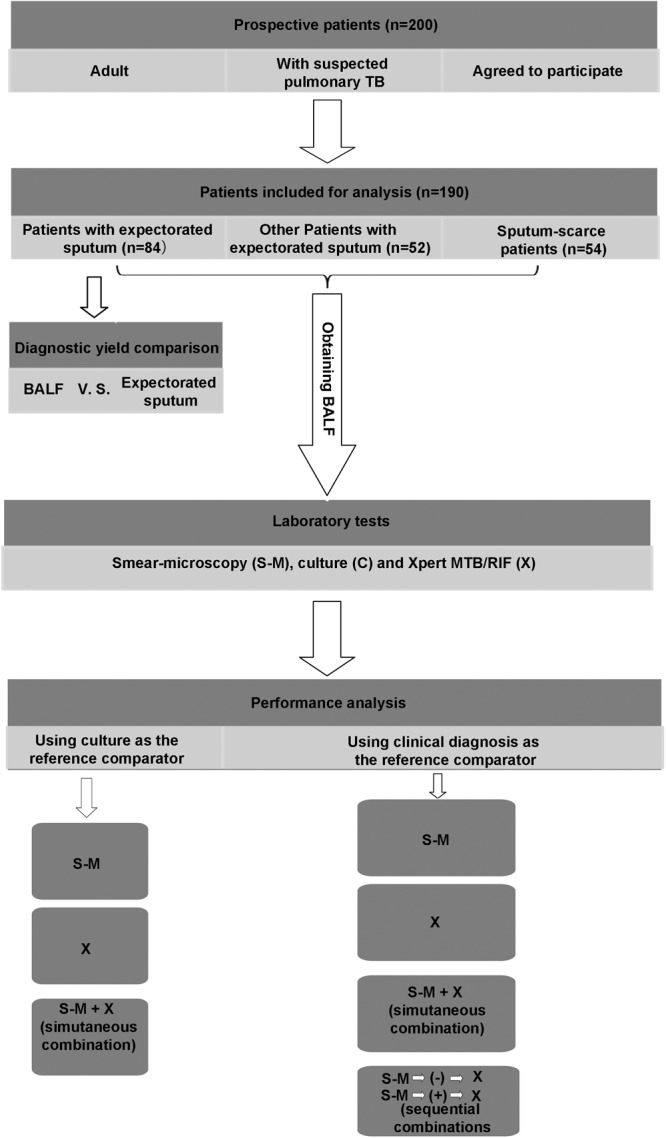
Patient flow through the study. TB, tuberculosis; BALF, bronchoalveolar lavage fluid; S-M, smear-microscopy; C, culture; X, Xpert MTB/RIF assay.

This prospective study was designed by following the general principle for the evaluation of diagnostic assays for infectious diseases recommended by The Special Program for Research and Training in Tropical Diseases (TDR) Diagnostics Evaluation Expert Panel (Banoo et al., 2010). The study evaluated 200 patients visiting the Wenzhou Central Hospital (WCH) and the Third Affiliated Hospital of Wenzhou Medical University (WMU) between November 2015 and December 2016 for suspected pulmonary TB. The WCH and the Third Affiliated Hospital are two teaching hospitals of WMU, China, covering a population of 11 million in South Zhejiang Province and North Fujian Province. Patients were enrolled in the study if they had chest computed tomography (CT) images suggesting possible pulmonary TB, and one of the following symptoms: Cough, chest tightness, chest pain, haemoptysis, fever, night sweats, loss of appetite, dyspnoea, and loss of weight. In addition to CT scan, all enrolled patients underwent a series of other clinical and laboratory examinations to assist diagnosis. These included physical examination, an interferon gamma release assay (IGRA) the T-SPOT TB test, bronchoscopy, histopathological examination of a bronchoalveolar biopsy specimen, staining and microscopy for acid-fast bacilli (smear-microscopy), liquid mycobacterial culture and Xpert MTB/RIF assay using BALF. The results were recorded on standardized case record forms. The Ethics Review Boards of WCH and the Third Affiliated Hospital of WMU both approved the study. Written informed consent for participation and the use of their data were provided to individual patients. The overall study was designed as described in **Figure 1**.

### Justification of Choosing BALF Over Expectorated Sputum for Evaluation of Xpert MTB/RIF

The quality of patient-collected sputum can vary due to any deficiency in the key steps of sputum-collection, including delivery of collection instructions by medical staff to patients, understanding of the requirements for proper sputum collection by patients and assessment of sputum quality by laboratory staff (Sakundarno et al., 2009). Chinese Center for Disease Control and Prevention (CCDC) guidelines for sputum collection requires expectorated sputum samples to be collected under the supervision of medical staff after patient orientation and education, and a quality evaluation to be performed by staff members at the diagnostic laboratories for all sputum samples received. Due to a large number of sputum samples required daily and the shortage of staff in the hospitals it has been difficult to vigorously follow the CCDC guideline when collecting sputum samples, possibly leading to inadequate quality of some sputum samples and a low diagnostic yield. A preliminary study was undertaken to compare the results of smear-microscopy and Xpert MTB/RIF obtained from expectorated sputum and BALF. Sputum specimens were obtained from 84 patients who were able to produce sputum, out of the 190 patients in the larger study. Staff members at the infectious diseases departments and diagnostic laboratories involved in sputum sample preparation were unaware of the experimental design. BALF was also obtained from the same 84 patients to compare the performance of the two specimen types when used for diagnostic assays.

### Bronchoscopy Procedure, Specimen Collection and Handling

Bronchoscopy was performed on all patients by experienced infectious disease physicians in dedicated suites under conscious sedation to collect BALF and biopsy samples. A bronchoscope was placed into an airway of an affected lung segment, as suggested by chest CT scan. Flexible bronchoscopes with a 2.0 mm diameter (model BF-Q170, Olympus Medical, Tokyo, Japan) or a 2.8 mm diameter (model BF-ITQ170, Olympus Medical, Tokyo, Japan) were used. After inspecting all visible segmental or bronchial trees, samples were collected from the lung segment or subsegment that showed abnormal lesions suggestive of active pulmonary TB on chest CT. For BALF collection, 50–100 mL of sterile saline was instilled and aspirated from the lung segments involved. Half of the specimen was used for smear-microscopy and mycobacterial culture and the other half was used for Xpert MTB/RIF tests (Lee et al., 2013). Histological examination was performed on transbronchial biopsies to assist diagnosis if necessary.

### Xpert MTB/RIF

The Xpert MTB/RIF assay was performed on sputum samples and BALF samples, following the manufacturer’s instructions. A total of 1 mL of BALF or sputum was transferred into the Xpert MTB/RIF cartridge without initial decontamination. Digestion solution (4% NaOH4 + 2% NaCl) was added and the cartridge was allowed to stand still for 15 min at room temperature, before placing the cartridge in the reactor of the Xpert MTB/RIF instrument for DNA extraction and PCR amplification. Xpert MTB/RIF software was used to interpret the results.

### Conventional Smear-Microscopy and Mycobacterial Culture Methods

Five milliliters of sputum and 1 mL of BALF were used for smear-microscopy and mycobacterial culture. Samples were pre-treated by decontamination with 4% weight/volume sodium hydroxide (NaOH) and centrifugation at 3000 × *g* for 20 min. The centrifuged deposit was stained by Ziehl–Neelsen and examined by microscopy (smear-microscopy), and cultured on Mycobacteria Growth Indicator Tube (MGIT, BD, Shanghai, China) for 2–8 weeks at 37°C. The acid fastness of the growth of mycobacteria in MGIT was verified by staining with Ziehl–Neelsen. A rapid immunochromatographic identification test, SD BIOLINE TB Ag MPT64 Rapid (Alere, Shanghai, China) was then carried out to differentiate *M. tuberculosis* complex from non-tuberculous mycobacteria (NTM). Mycobacterial growth from culture-positive MGIT also underwent routine phenotypic antibiotic susceptibility testing for rifampicin (R), isoniazid (H), streptomycin (S), and ethambutol (E), using BACTEC^TM^ MGIT^TM^ 960 SIRE KIT (BD, Shanghai, China) as per manufacturer’s instructions.

### Two Reference Standards for Diagnosis of Pulmonary TB

Two reference standards were used in this study for detection of *M. tuberculosis*. The first, the conventional “gold standard” mycobacterial culture (Theron et al., 2013; Luetkemeyer et al., 2016) was used to evaluate Xpert MTB/RIF, smear-microscopy or a combination of both. The second reference standard used was the final clinical diagnosis based on both clinical criteria including the patient’s response to anti-tuberculosis treatment as well as laboratory criteria. The clinical diagnosis was based on opinions of at least two qualified infectious disease physicians, using published two-step diagnostic criteria (Barnard et al., 2015). The initial criterion was a positive culture of *M. tuberculosis* complex from BALF. The secondary clinical case criteria were applied for culture-negative patients and included signs and symptoms compatible with active pulmonary TB; a positive T-SPOT. TB test result, a chest CT scan consistent with pulmonary TB, documented response to anti-TB treatment or non-treatment after 6 months indicated by a follow-up Chest CT scan. Such two-step diagnostic criteria were designed to predict the presence of active pulmonary TB and establish a credible reference standard, as the well-accepted high specificity (∼100%) of mycobacterial culture (the 1st criterion) allow very few “false-positive” cases (Lewinsohn et al., 2017), while the 2nd criterion further detects the “false-negative” cases in the studied population.

### Data Analysis and Statistical Methods

Data analysis was performed using SPSS 19.0 software (IBM Corp., Armonk, NY, United States) and Excel 2010 software (Microsoft Corp., Redmond, WA, United States). First, the sensitivity, specificity, accuracy, positive predictive values (PPV), and negative predictive values (NPV) of smear-microscopy, Xpert MTB/RIF or a combination of both were calculated, along with the corresponding 95% confidence intervals, obtained using the Wilson score binomial method (Luetkemeyer et al., 2016), with culture as the reference standard. Next, the same parameters were evaluated using the final clinical diagnosis as the reference. One-way analysis of variance (ANOVA) was used to determine whether there were any significant differences between the performance parameters of two assays or their combinations. Statistical significance was assumed at *p*-value of less than 0.05.

## Results

### Demographic and Clinical Characteristics of Participating Patients

Two hundred patients with suspected pulmonary TB were recruited from the two hospitals for this study. One hundred and ninety patients with a complete set of data necessary for analysis of the performance of Xpert MTB/RIF were selected for analysis. Demographic characteristics of selected patients are shown in **Table 1**.

**Table 1:** Demographic characteristics of participating patients.

Characteristics	No. (%)
Median age, years (range)	46.7 ± 16.6 (15–84)
Gender	
Male	120 (63.2)
Female	70 (36.8)
Patient status	
In patients	175 (92.1)
Outpatients	15 (7.9)
Clinical diagnosis	
Pulmonary tuberculosis	131 (68.9)
Non-mycobacterial infection	25 (13.2)
Previous pulmonary TB	18 (9.5)
Tubercular peritonitis	8 (4.2)
Non-tuberculous mycobacterial infection	3 (1.6)
Lung cancer	2 (1.1)
Lymphatic tuberculosis	1 (0.5)
Enterophthisis	1 (0.5)
Silicosis	1 (0.5)

*TB, tuberculosis*.

### Justification of Choosing BALF Over Expectorated Sputum for Evaluation of Xpert MTB/RIF

Patient-collected expectorated sputum had generally lower rates for detecting *M. tuberculosis* than BALF, irrespective of diagnostic assay employed (**Table 2**). On the basis of these results, it was decided to use BALF to compare the performance of Xpert MTB/RIF with that of conventional methods in diagnosing pulmonary TB.

**Table 2:** Comparison of BALF and expectorated sputum in detecting *Mycobacterium tuberculosis* in patients with a clinical diagnosis of pulmonary TB^a^.

	Number (%) of pulmonary TB patients (*N* = 44^b^) correctly diagnosed using	
	BALF	Expectorated sputum
Smear-microscopy	9 (20.5)	8 (18.2)
Xpert MTB/RIF	27 (61.4)	18 (40.9)

*^*a*^Clinical diagnosis was supported by positive culture, or/and clinical and radiological presentations consistent with active pulmonary TB infection, and documented improvement after anti-TB treatment. Refer to text for details.^*b*^44 out of 84 patients recruited were diagnosed with pulmonary TB. BALF, bronchoalveolar lavage fluid; TB, tuberculosis.*

### Performance of Xpert MTB/RIF Assay and Smear-Microscopy With Culture as the Reference Standard

Out of all 190 patients with suspected pulmonary TB, *M. tuberculosis* was detected from 77, 30, and 82 patients by mycobacterial culture, smear-microscopy, and Xpert MTB/RIF, respectively. **Table 3** summarizes the performance of Xpert MTB/RIF and smear-microscopy in the diagnosis of active pulmonary TB, with mycobacterial culture as the reference standard. Xpert MTB/RIF showed an overall higher accuracy than smear-microscopy (83.7 vs. 72.4%, *p* < 0.05), with a significantly higher sensitivity (83.1 vs. 37.7%, *p* < 0.05) but a slightly lower specificity (84.1 vs. 99.1%, *p* < 0.05). Combining smear-microscopy with Xpert MTB/RIF slightly increased its power to detect *M. tuberculosis*, with accuracy rising to 84.7%. However, using culture alone as the reference standard may not be adequate for evaluating Xpert MTB/RIF, since 54 of 131 (42.1%) cases of clinically diagnosed TB had negative culture results. Of these, 16 cases were further detected by Xpert MTB/RIF. We therefore used clinical diagnosis as the second reference standard to improve the evaluation of the Xpert MTB/RIF assay.

**Table 3:** Performance of smear-microscopy, Xpert MTB/RIF and their combination using culture as reference in detecting *M. tuberculosis*.

Diagnostic assay	Performance parameters [Mean percentage (N/N), 95% CI]				
	Sensitivity	Specificity	Accuracy	PPV	NPV
**Single tests**					
Smear-microscopy	37.7% (29/77) (26.0–49.4%)	99.1% (112/113) (91.2–100.0%)	74.2% (141/190) (64.7–83.7%)	96.7% (29/30) (66.7–100.0%)	70.0% (112/160) (64.4–75.6%)
Xpert MTB/RIF	83.1% (64/77) (70.1–96.1%)	84.1% (95/113) (75.2–92.9%)	83.7% (159/190) (73.2–94.2%)	78.1% (64/82) (65.9–90.2%)	88.0% (95/108) (78.7–97.2%)
**Combined tests^a^**	85.7% (66/77) (72.7–100.0%)	84.1% (95/113) (75.2–93.8%)	84.7% (161/190) (74.2–96.3%)	78.6% (66/84) (66.7–91.7%)	89.6% (95/106) (80.2–100.0%)

*BALF was used for testing. CI, confidence interval; PPV, positive predictive value; NPV, negative predictive value. ^*a*^Results of smear-microscopy and Xpert MTB/RIF were combined. Negative results of both assays were considered as a “negative” and any positive was taken as a “positive” for the combined assays*.

### Performance of Xpert MTB/RIF and Conventional Methods With Clinical Diagnosis as the Reference Comparator

**Table 4** summarizes the overall performance of Xpert MTB/RIF and smear-microscopy as single tests and combined tests in the diagnosis of pulmonary TB, using the clinical diagnosis as the reference comparator. As a single test, Xpert MTB/RIF showed high accuracy of 72.1%, supported by a sensitivity of 61.1%, NPV of 52.8%, specificity of 96.6% and PPV of 97.6%. Smear-microscopy had a significantly lower sensitivity (22.9%) and a slightly but insignificantly higher specificity (100%). We further examined the performance of combinations of Xpert MTB/RIF and smear-microscopy in a simultaneous or sequential order. Simultaneously combining Xpert MTB/RIF with smear-microscopy, compared with smear-microscopy or Xpert MTB/RIF alone increased assay sensitivities by 39.7 and 1.5%, respectively (**Table 4**). Sequential combinations of smear-microscopy with Xpert MTB/RIF showed that Xpert MTB/RIF maintained a high power to detect *M. tuberculosis* in pulmonary TB patients who failed smear-microscopy screening, with a sensitivity of 51.5%, NPV of 53.8%, a specificity of 96.6%, and PPV of 96.3% (**Table 4**). A more detailed decomposition analysis was performed to compare the power of each assay in identifying pulmonary TB cases missed by others.

**Table 4:** Performance of smear-microscopy, Xpert MTB/RIF and their combinations using clinical diagnosis as reference in detecting *M. tuberculosis*.

Diagnostic assay	Performance parameters [Mean percentage (N/N), 95% CI]				
	Sensitivity	Specificity	Accuracy	PPV	NPV
**Single tests**					
Smear-microscopy	22.9% (30/131) (16.0–29.8%)	100.0% (59/59) (84.8–100.0%)	46.8% (89/190) (37.4–56.3%)	100.0% (30/30) (70.0–100.0%)	36.9% (59/160) (31.3–42.5%)
Xpert MTB/RIF	61.1% (80/131) (51.2–71.0%)	96.6% (57/59) (74.6–100.0%)	72.1% (137/190) (58.42–85.8%)	97.6% (80/82) (81.7–100.0%)	52.8% (57/108) (40.7–64.8%)
**Simultaneous combination^a^**					
Smear-microscopy + Xpert MTB/RIF	62.6% (82/131) (52.7–72.5%)	96.6% (57/59) (74.6–100.0%)	73.2% (139/190) (59.5–86.8%)	97.6% (82/84) (82.1–100.0%)	53.8% (57/106) (41.5–66.0%)
**Sequential combination^b^**					
Smear-microscopy (-) + Xpert MTB/RIF	51.5% (52/101) (39.6–62.4%)	96.6% (57/59) (76.3–100.0%)	68.1% (109/160) (53.1–81.9%)	96.3% (52/54) (74.1–100.0%)	53.8% (57/106) (42.5–64.2%)
Smear-microscopy (+) + Xpert MTB/RIF	93.3% (28/30) (83.3–100%)	N/A (0/0)	93.3% (28/30) (83.33-100%)	100% (28/28) (89.29-100%)	N/A (0/2)

*BALF was used for testing. CI, Confidence interval; PPV, Positive predictive value; NPV, Negative predictive value; N/A, Not available. ^*a*^Simultaneous combination: Results of smear-microscopy and Xpert MTB/RIF were combined. Both negative was considered as a “negative” and any positive was taken as a “positive.”*

*^*b*^Sequential combination: Xpert MTB/RIF assay performed for pulmonary TB patients with negative (-) or positive (+) smear-microscopy results, respectively*

### Capability of Xpert MTB/RIF to Detect Clinically Diagnosed Pulmonary TB That Generated Negative Results in Conventional Assays

Xpert MTB/RIF assay supported the clinical diagnosis in 52 out of 101 (51.5%) smear-negative TB patients and 16 out of 54 (29.6%) culture-negative patients. In contrast, smear-microscopy only detected 1 out of 54 TB patients with negative culture results and 2 out of 52 patients with negative Xpert MTB/RIF results. Mycobacterial culture detected 13 out of 51 patients (25.5%) with negative Xpert MTB/RIF results and 48 out of 101 patients (47.5%) with negative smear-microscopy results, suggesting complementary roles between mycobacterial culture and Xpert MTB/RIF in detecting pulmonary *M. tuberculosis*. Three patients with NTM infection were identified only by the culture method. Twenty patients were clinically diagnosed with previous pulmonary TB, among them two were Xpert MTB/RIF positive, albeit with ultralow readings. Follow-up chest CT scan confirmed the initial clinical diagnosis.

### Xpert MTB/RIF for the Detection of Anti-TB Drug-Resistance

Although the Xpert MTB/RIF assay and conventional susceptibility testing gave most consistent results, there were a few important differences. The majority of *M. tuberculosis* isolates detected simultaneously by mycobacterial culture and the Xpert MTB/RIF assay (58 out of 62) were susceptible to first-line anti-TB drugs tested: H, R, S, and E, according to the Xpert MTB/RIF assay. Resistance to R was reported for four patients by the X-pert MDR/RIF assay. Parallel conventional antibiotic susceptibility tests using *M. tuberculosis* growth in MGIT showed one isolate was resistant to H/R/S/E, one was resistant to H/R/S, one was resistant to H only, and the last one was susceptible to all anti-TB drugs tested. Among the 58 patients with negative MTB/RIF readings, MGIT growth for two patients showed increased resistance to isoniazid and ethambutol, respectively.

## Discussion

In this study, we performed a rigorous, standardized, head-to-head comparison of the performance of the Xpert MTB/RIF assay with conventional laboratory methods for diagnosis of adult pulmonary TB, using two different reference standards and with BALF obtained from adult Chinese patients. Key findings of this study include: BALF outperformed expectorated sputum collected under current practice in these two hospitals; Xpert MTB/RIF with BALF was found to be an ideal single-assay combination for rapid and accurate diagnosis of pulmonary TB; a combination of smear-microscopy and Xpert MTB/RIF remained an accurate early predictor for pulmonary TB. Predictions by the Xpert MTB/RIF assay for multidrug-resistance of *M. tuberculosis* require further improvement.

Various samples have been used for TB diagnosis, including spontaneously expectorated sputum, induced sputum, tracheal aspirate, pleural fluid, cerebral spinal fluid, pericardial fluid, biopsy, nasopharyngeal aspiration, gastric lavage, urine, cold abscess aspirate, and BALF (Hepple et al., 2012; Bates et al., 2013; Mathew et al., 2014; Pang et al., 2014; Zhang et al., 2016). Although different opinions have been expressed regarding the importance of the source and type of test sample (Bunyasi et al., 2015), the quality of the specimen has been considered as a determinant factor affecting assay performance (Pinto and Udwadia, 2013). Theron et al. (2014) found that expectorated sputum was the best specimen with the highest sensitivity among all pulmonary specimens including BALF, possibly due to the highest bacterial load of the sputum that has been expectorated properly (Iyer et al., 2011; Theron et al., 2014). However, unacceptable quality of sputum samples received by diagnostic laboratories is a common issue in China and worldwide and has become a significant cause of low diagnostic yield (Heineman et al., 1977; Macq et al., 2005; Ou et al., 2015). We suspected that the current inadequacies in sputum collection in the two hospitals that participated in this study might have led to insufficient quality of expectorated sputum used for testing and consequentially may have affected the performance of all diagnostic assays. In the two hospitals, bronchoscopy and BALF are routinely recommended for patients who are sputum-scarce or have negative sputum smear-microscopy and Xpert MTB/RIF, while their clinical and radiological examinations are highly suggestive of pulmonary TB, or for differential diagnosis to clarify the underlying cause of a radiographic abnormality (de Gracia et al., 1988; Iyer et al., 2011). When a head-to-head comparison was carried out between BALF and expectorated sputum obtained from the same patients at the same time point to avoid sampling bias, BALF was found to be superior to expectorated sputum in diagnosing pulmonary TB in the population sampled.

The diagnostic performance of X-pert MTB/RIF, using different samples including BALF, has been assessed by many studies in adult patients from various geographical regions, including low-TB prevalence countries such as the United States and higher TB prevalence countries such as South Korea, Brazil, and South Africa (Lee et al., 2013; Theron et al., 2013; Le Palud et al., 2014; Luetkemeyer et al., 2016). Although this assay combined with BALF has been evaluated in children in China, it has not been assessed in adult local patients. Information on the performance of Xpert MTB/RIF combined with BALF in adult Chinese patients would facilitate the integration of this assay into current tuberculosis diagnostic algorithms. In our prospective study, we found high sensitivities (83.1 and 61.1%), specificities (84.1 and 96.6%), PPV (78.1 and 97.6%), and NPV (88.0 and 52.8%) of Xpert MTB/RIF in detecting pulmonary TB, using the conventional “gold-standard” mycobacterial culture and the more clinically relevant final diagnosis as reference comparators, respectively. Similar sensitivities, specificities, PPVs and NPVs were reported by Lee et al. (2013) in a recently published retrospective study using BALF from South Korean patients and culture as the reference standard. Our finding is also in line with other reports from South Africa using Xpert MTB/RIF on BALF and other respiratory samples, with sensitivities ranging from 60 to 96% and specificities ranging from 50 to 100% (Ciftci et al., 2011; Lee et al., 2013; Theron et al., 2013; Le Palud et al., 2014; Walters et al., 2014; Detjen et al., 2015). In the present study, fifty-one clinical cases of pulmonary TB showed false-negative readings for the Xpert MTB/RIF assay. This may be explained by a low mycobacterial burden of these cases (Lee et al., 2013), since a minimum number of 131 mycobacterial colony forming units is required to be present in the specimen to produce a positive Xpert MTB/RIF reading (Bunyasi et al., 2015). Falsely positive results are another major concern for using RT-PCR-based Xpert MTB/RIF for TB diagnosis, which is often related to the presence of dead *M. tuberculosis* cells after anti-TB treatment or a previous pulmonary TB status (Friedrich et al., 2013). In our study, among 18 patients diagnosed with previous pulmonary TB, 16 remained negative to Xpert MTB/RIF and two were positive. Detecting *M. tuberculosis* DNA by Xpert MTB/RIF from these two patients, who received no anti-TB treatment and gave negative results in a 6-month follow-up CT scan has resulted in a lower specificity of Xpert MTB/RIF, relative to that of smear-microscopy (**Table 3**). Smear-microscopy, the frontline diagnostic test currently used in China, showed a very low sensitivity in detecting *M. tuberculosis* from BALF. Xpert MTB/RIF demonstrated a high sensitivity for these smear-microscopy negative TB cases, as shown in the current study and also other studies (51.5% in our study and 49 to 72% in other studies) (Bates et al., 2013; Jafari et al., 2013; Pang et al., 2014; Yin et al., 2014; Singh et al., 2016). Furthermore, Xpert MTB/RIF detected approximately 29.6% of culture-negative cases, which is higher than reported in the literature [14% (Walters et al., 2014), 12.5% (Barnard et al., 2015; Celik et al., 2015; Heidebrecht et al., 2016), 7% (Barnard et al., 2015)]. However, it is important to note that mycobacterial culture, the conventional “gold standard,” is still a powerful and complementary tool in confirming pulmonary TB cases that are misdiagnosed by smear-microscopy or Xpert MTB/RIF; it also remains the irreplaceable method for the detection of NTM.

With clinical diagnosis as a reference standard with higher clinical reliability compared to culture, a combination of smear-microscopy and Xpert MTB/RIF for early diagnosis of TB outperformed all single tests in the context of accuracy. A potential major concern of using Xpert MTB/RIF as a single test or in combination with other assays is the high cost. Though a single Xpert MTB/RIF kit costs less than 10 dollars, this procedure is currently priced at approximately 80 dollars in China without medicare rebate. More than 70 million dollars would be required for an accurate early screening of the 918,000 new TB patients in China using this assay (WHO, 2017). There is an urgent need to accomonadate Xpert MTB/RIF into the current Chinese TB Control Program and to lower the cost of Xpert MTB/RIF. Recent cost-benefit analysis of Xpert MTB/RIF for suspected TB cases found using this assay as an adjunct for smear-microscopy for early diagnosis actually reduced expenditures in pulmonary TB patients (Theron et al., 2012; Diel et al., 2016).

The incidence of rifampicin resistance was found to be low in our study, consistent with the findings from previous studies (Lee et al., 2013; Theron et al., 2013; Le Palud et al., 2014; Luetkemeyer et al., 2016). We also found that rifampicin resistance reported by Xpert MTB/RIF could not fully represent the multidrug resistance profile demonstrated by conventional antibiotic susceptibility testing. However, no solid conclusion can be drawn due to the fact that very few rifampicin-resistant TB cases were identified in this study and mutations of the *rpo*B gene suggested by Xpert MTB/RIF have not been further examined by DNA sequencing, which is a limitation. Discrepancy between the positive rifampicin-resistance detected by Xpert RIF/MTB, and the negative phenotype using conventional susceptibility testing could be explained by the presence of “disputed” *rpoB* mutations, or a silent mutation in the *rpoB* gene (Van Deun et al., 2013; Mathys et al., 2014). Failure to clarify the discrepancy by identifying the mutation in the *rpo*B gene often results in a poor treatment outcome (Van Deun et al., 2013; Mathys et al., 2014). A study with a larger sample size and incorporating DNA sequencing for *rpo*B mutations is needed for a comprehensive assessment of the performance of Xpert MTB/RIF in detecting rifampicin/multidrug resistance in adult Chinese patients, with more accurate interpretation of rifampicin resistance detected by Xpert MTB/RIF.

The prospective nature of this study allowed us to implement Xpert MTB/RIF, smear-microscopy and culture on aliquots of the same specimen, ensuring a valid comparison without a sample bias caused by the heterogeneity of specimens (Theron et al., 2013). Another advantage of this prospective study was the access to a complete set of data necessary for a systemic and comprehensive analysis of the performance of diagnostic assays. Unlike many other studies that only used culture as the reference comparator, we employed clinical diagnosis as the second and more powerful reference standard for performance analysis. Culture was considered as a highly deficient “gold standard” for TB diagnosis and might be inadequate for assessing the accuracy of molecular diagnostic assays that have a similar sensitivity to culture [(Banoo et al., 2010; Theron et al., 2013; Barnard et al., 2015; Bunyasi et al., 2015; Ho et al., 2016; Singh et al., 2016) and this study]. Previous studies using reference standards other than culture, with additional criteria that lead to a positive clinical diagnosis, have often shown a higher predictive value and specificity of Xpert MTB/RIF than that reported by studies using culture as reference standard (Theron et al., 2011; Ho et al., 2016; Walters et al., 2017). A major limitation of the present study is the relatively small patient size and the short (13-month) study duration. The performance of the Xpert MTB/RIF assay combined with BALF for the detection of pulmonary TB in adult Chinese patients might be different in larger cohorts or in studies performed over a longer period. Another limitation of the current study is the use of the first generation of Xpert MTB/RIF. As reported by many others and also found in this study, Xpert MTB/RIF has relatively lower sensitivity with smear-negative pulmonary samples (Detjen et al., 2015). The newly developed Xpert Ultra assay has demonstrated a significant improvement in TB and rifampicin-resistance detection, with higher sensitivities on both total sputum samples and smear-microscopy negative sputum samples (Chakravorty et al., 2017). This kit, however, is only currently accessible for very few research institutes but not the majority of public hospitals in China.

## Conclusion

In summary, our study provides a solid foundation for the potential integration of Xpert MTB/RIF into the current diagnostic algorithms for pulmonary TB in China. The Xpert MTB/RIF assay using BALF offers obvious advantages over conventional tests. As a single test, it has a much higher sensitivity than smear-microscopy, and a much shorter turnaround time compared with mycobacterial culture. Combing smear-microscopy and Xpert MTB/RIF appears to be an accurate and cost-effective tool for the early diagnosis of pulmonary TB. A combination of these two assays for early diagnosis and culture for later confirmation can be used as a rationalized diagnostic algorithm in Chinese tertiary hospitals. Integrating X-pert MTB/RIF into the current TB diagnostic pipeline in China does increase the cost, for example, by several times for pulmonary TB patients with negative smear-microscopy results. The higher cost, however, can be compensated by its higher accuracy, short turnaround time, and the less treatment cost spent on multidrug resistant patients and patients with uncertain diagnosis (Theron et al., 2013; Diel et al., 2016).

## Author Contributions

SY, LH, FS, YQ, and XP designed the project. SY and LH recruited the patients and performed the bronchoscopy. FS, XP, LH, SY, YQ, and MD interpreted the data. FS, YQ, and MD wrote the main manuscript text. SY, XP, and LH revised the main manuscript. All authors reviewed and approved the manuscript.

## Conflict of Interest Statement

The authors declare that the research was conducted in the absence of any commercial or financial relationships that could be construed as a potential conflict of interest.

## References

[B1] Banoo S. Bell D. Bossuyt P. Herring A. Mabey D. Poole F. (2010). Evaluation of diagnostic tests for infectious diseases: general principles. *Nat. Rev. Microbiol.* 8 S17–S29. 21548184

[B2] Barnard D. A. Irusen E. M. Bruwer J. W. Plekker D. Whitelaw A. C. Deetlefs J. D. (2015). The utility of Xpert MTB/RIF performed on bronchial washings obtained in patients with suspected pulmonary tuberculosis in a high prevalence setting. *BMC Pulm. Med.* 15:103. 10.1186/s12890-015-0086-z 26377395 PMC4573925

[B3] Bates M. O’grady J. Maeurer M. Tembo J. Chilukutu L. Chabala C. (2013). Assessment of the Xpert MTB/RIF assay for diagnosis of tuberculosis with gastric lavage aspirates in children in sub-Saharan Africa: a prospective descriptive study. *Lancet Infect. Dis.* 13 36–42. 10.1016/S1473-3099(12)70245-1 23134697

[B4] Bunyasi E. W. Tameris M. Geldenhuys H. Schmidt B. M. Luabeya A. K. Mulenga H. (2015). Evaluation of Xpert(R) MTB/RIF assay in induced sputum and gastric lavage samples from young children with suspected tuberculosis from the MVA85A TB vaccine trial. *PLoS One* 10:e0141623. 10.1371/journal.pone.0141623 26554383 PMC4640848

[B5] Celik C. Gozel M. G. Bakici M. Z. Berk S. Ozsahin S. L. Gulturk E. (2015). Applicability of Xpert MTB/RIF assay for routine diagnosis of tuberculosis: a four-year single-center experience. *Turk. J. Med. Sci.* 45 1329–1334. 10.3906/sag-1407-56 26775391

[B6] Chakravorty S. Simmons A. M. Rowneki M. Parmar H. Cao Y. Ryan J. (2017). The new Xpert MTB/RIF Ultra: improving detection of *Mycobacterium tuberculosis* and resistance to rifampin in an assay suitable for point-of-care testing. *mBio* 8:e00812-17. 10.1128/mBio.00812-17 28851844 PMC5574709

[B7] Ciftci I. H. Aslan M. H. Asik G. (2011). Evaluation of Xpert MTB/RIF results for the detection of *Mycobacterium tuberculosis* in clinical samples. *Mikrobiyol. Bul.* 45 43–47. 21341158

[B8] de Gracia J. Curull V. Vidal R. Riba A. Orriols R. Martin N. (1988). Diagnostic value of bronchoalveolar lavage in suspected pulmonary tuberculosis. *Chest* 93 329–332. 10.1378/chest.93.2.329 3123151

[B9] Detjen A. K. Dinardo A. R. Leyden J. Steingart K. R. Menzies D. Schiller I. (2015). Xpert MTB/RIF assay for the diagnosis of pulmonary tuberculosis in children: a systematic review and meta-analysis. *Lancet Respir. Med.* 3 451–461. 10.1016/S2213-2600(15)00095-8 25812968 PMC4756280

[B10] Diel R. Nienhaus A. Hillemann D. Richter E. (2016). Cost-benefit analysis of Xpert MTB/RIF for tuberculosis suspects in German hospitals. *Eur. Respir. J.* 47 575–587. 10.1183/13993003.01333-2015 26647440

[B11] Friedrich S. O. Rachow A. Saathoff E. Singh K. Mangu C. D. Dawson R. (2013). Assessment of the sensitivity and specificity of Xpert MTB/RIF assay as an early sputum biomarker of response to tuberculosis treatment. *Lancet Respir. Med.* 1 462–470. 10.1016/S2213-2600(13)70119-X 24429244

[B12] Heidebrecht C. L. Podewils L. J. Pym A. S. Cohen T. Mthiyane T. Wilson D. (2016). Assessing the utility of Xpert^®^ MTB/RIF as a screening tool for patients admitted to medical wards in South Africa. *Sci. Rep.* 6:19391. 10.1038/srep19391 26786396 PMC4726405

[B13] Heineman H. S. Chawla J. K. Lopton W. M. (1977). Misinformation from sputum cultures without microscopic examination. *J. Clin. Microbiol.* 6 518–527. 336644 10.1128/jcm.6.5.518-527.1977 PMC274808

[B14] Hepple P. Ford N. Mcnerney R. (2012). Microscopy compared to culture for the diagnosis of tuberculosis in induced sputum samples: a systematic review. *Int. J. Tuberc. Lung Dis.* 16 579–588. 10.5588/ijtld.11.0617 22410498

[B15] Hill P. C. Whalen C. C. (2015). Prevalence of tuberculosis in China. *Lancet* 385:773 10.1016/S0140-6736(15)60436-6 PMC6563328 25752176

[B16] Ho J. Nguyen P. T. Nguyen T. A. Tran K. H. Van Nguyen S. Nguyen N. V. (2016). Reassessment of the positive predictive value and specificity of Xpert MTB/RIF: a diagnostic accuracy study in the context of community-wide screening for tuberculosis. *Lancet Infect. Dis.* 16 1045–1051. 10.1016/S1473-3099(16)30067-6 27289387

[B17] Iyer V. N. Joshi A. Y. Boyce T. G. Brutinel M. W. Scalcini M. C. Wilson J. W. (2011). Bronchoscopy in suspected pulmonary TB with negative induced-sputum smear and MTD((R)) Gen-probe testing. *Respir. Med.* 105 1084–1090. 10.1016/j.rmed.2011.03.003 21420844

[B18] Jafari C. Ernst M. Kalsdorf B. Lange C. (2013). Comparison of molecular and immunological methods for the rapid diagnosis of smear-negative tuberculosis. *Int. J. Tuberc. Lung Dis.* 17 1459–1465. 10.5588/ijtld.13.0108 24125451

[B19] Le Palud P. Cattoir V. Malbruny B. Magnier R. Campbell K. Oulkhouir Y. (2014). Retrospective observational study of diagnostic accuracy of the Xpert(R) MTB/RIF assay on fiberoptic bronchoscopy sampling for early diagnosis of smear-negative or sputum-scarce patients with suspected tuberculosis. *BMC Pulm. Med.* 14:137. 10.1186/1471-2466-14-137 25115239 PMC4137109

[B20] Lee H. Y. Seong M. W. Park S. S. Hwang S. S. Lee J. Park Y. S. (2013). Diagnostic accuracy of Xpert^®^ MTB/RIF on bronchoscopy specimens in patients with suspected pulmonary tuberculosis. *Int. J. Tuberc. Lung Dis.* 17 917–921. 10.5588/ijtld.12.0885 23621953

[B21] Lewinsohn D. M. Leonard M. K. Lobue P. A. Cohn D. L. Daley C. L. Desmond E. (2017). Official American thoracic society/infectious diseases society of America/centers for disease control and prevention clinical practice guidelines: diagnosis of tuberculosis in adults and children. *Clin. Infect. Dis.* 64 111–115. 10.1093/cid/ciw778 28052967 PMC5504475

[B22] Luetkemeyer A. F. Firnhaber C. Kendall M. A. Wu X. Mazurek G. H. Benator D. A. (2016). Evaluation of Xpert MTB/RIF versus AFB smear and culture to identify pulmonary tuberculosis in patients with suspected tuberculosis from low and higher prevalence settings. *Clin. Infect. Dis.* 62 1081–1088. 10.1093/cid/ciw035 26839383 PMC4826450

[B23] Macq J. Solis A. Velazquez H. Dujardin B. (2005). Informing the TB suspect for sputum sample collection and communicating laboratory results in Nicaragua: a neglected process in tuberculosis case finding. *Salud Publica Mex.* 47 303–307. 10.1590/S0036-36342005000400008 16259292

[B24] Mathew J. L. Vijayasekharan D. Singh S. (2014). Is Xpert MTB/RIF assay in gastric lavage aspirate useful for diagnosis of smear-negative childhood pulmonary tuberculosis? *Indian Pediatr.* 51 1007–1011. 10.1007/s13312-014-0548-z 25560161

[B25] Mathys V. Van De Vyvere M. De Droogh E. Soetaert K. Groenen G. (2014). False-positive rifampicin resistance on Xpert(R) MTB/RIF caused by a silent mutation in the rpoB gene. *Int. J. Tuberc. Lung Dis.* 18 1255–1257. 10.5588/ijtld.14.0297 25216843

[B26] Meyer A. J. Atuheire C. Worodria W. Kizito S. Katamba A. Sanyu I. (2017). Sputum quality and diagnostic performance of GeneXpert MTB/RIF among smear-negative adults with presumed tuberculosis in Uganda. *PLoS One* 12:e0180572. 10.1371/journal.pone.0180572 28686705 PMC5501569

[B27] Ou X. Xia H. Li Q. Pang Y. Wang S. Zhao B. (2015). A feasibility study of the Xpert MTB/RIF test at the peripheral level laboratory in China. *Int. J. Infect. Dis.* 31 41–46. 10.1016/j.ijid.2014.09.011 25447720

[B28] Pang Y. Wang Y. Zhao S. Liu J. Zhao Y. Li H. (2014). Evaluation of the Xpert MTB/RIF assay in gastric lavage aspirates for diagnosis of smear-negative childhood pulmonary tuberculosis. *Pediatr. Infect. Dis. J.* 33 1047–1051. 10.1097/INF.0000000000000403 25361186

[B29] Pinto L. M. Udwadia Z. F. (2013). Xpert MTB/RIF and pulmonary tuberculosis: time to delve deeper? *Thorax* 68 987–988. 10.1136/thoraxjnl-2013-203885 23828122

[B30] Sakundarno M. Nurjazuli N. Jati S. P. Sariningdyah R. Purwadi S. Alisjahbana B. (2009). Insufficient quality of sputum submitted for tuberculosis diagnosis and associated factors, in Klaten district, Indonesia. *BMC Pulm. Med.* 9:16. 10.1186/1471-2466-9-16 19426477 PMC2689165

[B31] Singh M. Sethi G. R. Mantan M. Khanna A. Hanif M. (2016). Xpert((R)) MTB/RIF assay for the diagnosis of pulmonary tuberculosis in children. *Int. J. Tuberc. Lung Dis.* 20 839–843. 10.5588/ijtld.15.0824 27155190

[B32] Theron G. Peter J. Calligaro G. Meldau R. Hanrahan C. Khalfey H. (2014). Determinants of PCR performance (Xpert MTB/RIF), including bacterial load and inhibition, for TB diagnosis using specimens from different body compartments. *Sci. Rep.* 4:5658. 10.1038/srep05658 25014250 PMC5375978

[B33] Theron G. Peter J. Meldau R. Khalfey H. Gina P. Matinyena B. (2013). Accuracy and impact of Xpert MTB/RIF for the diagnosis of smear-negative or sputum-scarce tuberculosis using bronchoalveolar lavage fluid. *Thorax* 68 1043–1051. 10.1136/thoraxjnl-2013-203485 23811536 PMC5523966

[B34] Theron G. Peter J. Van Zyl-Smit R. Mishra H. Streicher E. Murray S. (2011). Evaluation of the Xpert MTB/RIF assay for the diagnosis of pulmonary tuberculosis in a high HIV prevalence setting. *Am. J. Respir. Crit. Care Med.* 184 132–140. 10.1164/rccm.201101-0056OC 21493734

[B35] Theron G. Pooran A. Peter J. Van Zyl-Smit R. Kumar Mishra H. Meldau R. (2012). Do adjunct tuberculosis tests, when combined with Xpert MTB/RIF, improve accuracy and the cost of diagnosis in a resource-poor setting? *Eur. Respir. J.* 40 161–168. 10.1183/09031936.00145511 22075479 PMC5523948

[B36] Van Deun A. Aung K. J. Bola V. Lebeke R. Hossain M. A. De Rijk W. B. (2013). Rifampin drug resistance tests for tuberculosis: challenging the gold standard. *J. Clin. Microbiol.* 51 2633–2640. 10.1128/JCM.00553-13 23761144 PMC3719626

[B37] Walters E. Demers A. M. Van Der Zalm M. M. Whitelaw A. Palmer M. Bosch C. (2017). Stool culture for diagnosis of pulmonary tuberculosis in children. *J. Clin. Microbiol.* 33 3355–3365. 10.1128/JCM.00801-17 28904186 PMC5703802

[B38] Walters E. Goussard P. Bosch C. Hesseling A. C. Gie R. P. (2014). GeneXpert MTB/RIF on bronchoalveolar lavage samples in children with suspected complicated intrathoracic tuberculosis: a pilot study. *Pediatr. Pulmonol.* 49 1133–1137. 10.1002/ppul.22970 24339262

[B39] WHO (2017). *Global Tuberculosis Report* 2016. Geneva: WHO.

[B40] Wu B. Yu Y. Xie W. Liu Y. Zhang Y. Hu D. (2017). Epidemiology of tuberculosis in Chongqing, China: a secular trend from 1992 to 2015. *Sci. Rep.* 7:7832. 10.1038/s41598-017-07959-2 28798367 PMC5552739

[B41] Yin Q. Q. Jiao W. W. Han R. Jiao A. X. Sun L. Tian J. L. (2014). Rapid diagnosis of childhood pulmonary tuberculosis by Xpert MTB/RIF assay using bronchoalveolar lavage fluid. *Biomed. Res. Int.* 2014:310194. 10.1155/2014/310194 25165698 PMC4140106

[B42] Zhang A. M. Li F. Liu X. H. Xia L. Lu S. H. (2016). Application of Gene Xpert Mycobacterium tuberculosis DNA and resistance to rifampicin assay in the rapid detection of tuberculosis in children. *Zhonghua Er Ke Za Zhi* 54 370–374. 10.3760/cma.j.issn.0578-1310.2016.05.012 27143080

